# The Effectiveness and Safety of a Nutraceutical Combination in Overweight Patients with Metabolic Syndrome

**DOI:** 10.3390/nu16233977

**Published:** 2024-11-21

**Authors:** Lucilla Ricottini, Sabrina Basciani, Maria Letizia Spizzichini, Domenico de Mattia, Manuela Coniglio-Iannuzzi delle Noci, Sasha Sorrentino, Maurizio Nordio

**Affiliations:** 1 Sinergheia Clinical Center, 00192 Rome, Italy; 2 Department of Experimental Medicine, University “Sapienza”, 00161 Rome, Italy; 3 De Mattia Center for Nutrition and Obesity, 70124 Bari, Italy; 4 Delle Noci Multy Specialty Center, 70124 Bari, Italy; 5 Villa Brasini Medical Center, 20100 Milan, Italy

**Keywords:** insulin resistance, overweight, metabolic dysfunction, nutraceuticals

## Abstract

Background: The aim of the present study was to evaluate the effectiveness and safety of a nutraceutical combination given to insulin-resistant overweight patients with altered lipid profiles. To this end, an observational study was designed in which 74 individuals (50 females and 24 males) underwent an observational period of 3 months. Methods: During this time, a specific nutraceutical combination containing myo-inositol, glycine, *Coprinus comatus*, α-lipoic acid, phlorizin, zinc, vitamin B^6^, and chromium picolinate was administered. Patients were asked not to modify their lifestyles so that no variable that might interfere with results was introduced. Results: After the 3-month period, the obtained data revealed that insulin levels significantly decreased with respect to the baseline, while glucose levels exhibited a trend towards lower concentrations, which was not significant. In addition, HOMA-IR index, body weight, BMI, and abdominal circumference values all decreased significantly. Regarding lipid profiles, the data obtained before and after the 3-month period showed statistically significant decreases in concentrations of total cholesterol, LDL cholesterol, and triglyceride, as well as a small but statistically significant concomitant increase in HDL cholesterol. Conclusions: Thus, on the basis of these data, it may be stated that the specific nutraceutical combination used in the present study significantly ameliorated a number of metabolic parameters without measurable side effects. The efficacy and safety of the product were, therefore, confirmed in our group of patients.

## 1. Introduction

The quest for the best strategy to apply in cases of overweight, obesity, metabolic alterations, and increased cardiovascular risk has challenged scientists for decades. This is due, at least in part, to our ability to store energy without limits, even if it is not needed, as well as our unrestricted access to commercially available food sources. In addition, the promotion of nutritionally unhealthy meals by food industries has induced the population to consume fewer fats and more carbohydrates. This has contributed to an increased incidence of overweight, inflammation, and metabolic disruption, with all their well-known consequences. It is now predicted that, by 2050, the prevalence of type 2 diabetes mellitus (T2DM) will rise dramatically and that the mortality risk for all causes will increase in proportion with increases in body mass index (BMI) [1,2]. These developments are likely to have a huge economic impact, with increased burdens on public health systems worldwide.

Today, it is widely known that tackling medical problems due to overweight and/or obesity is extremely difficult, especially over the long term, due to the complexity of the issue. It is universally accepted that lifestyle changes (appropriate nutritional intervention and physical activity) are essential if the path to comorbidities is to be reversed; however, it is often difficult to convince an individual to make changes to their lifestyle and, more importantly, to maintain these changes indefinitely [3]. For this reason, it is necessary to adopt a multidisciplinary approach, mainly to control metabolic dysregulation and cardiovascular issues; this may include the use of adjunctive therapies involving either nutraceuticals and/or pharmacological drugs, with additional psychological interventions in some cases.

The use of nutraceuticals for therapeutic purposes has gained considerable attention in recent years. This is due to an increasing body of evidence from around the world that demonstrates their effectiveness in cases of metabolic disturbances, re-balancing inflammation, gut microbiota, insulin resistance, and dyslipidemia, among others [4]. In addition, patients tend to prefer using nutraceutical products as opposed to drugs. This is not only because nutraceuticals are associated with lower levels of side effects but also because of a subjective perception that nutraceutical products are “safer” and more tolerable.

For this reason, many nutraceutical products are now at the disposal of doctors, particularly those dealing with cases of overweight, obesity, metabolic disturbances, pre-diabetes, and dyslipidemia. For the present study, a particular combination of nutraceutical products was chosen for evaluation due to the known ability of each component ingredient to act in a way that ameliorates metabolic imbalance and/or decreases levels of inflammation.

Among these, myo-inositol, which belongs to the inositol family, is a substance whose ability to reduce insulin resistance and inflammation is well known [1]. Myo-inositol plays two different roles in organisms. As a part of cell membranes, it is involved in intracellular signal transduction through the phosphatidylinositol pathway. It is also involved in the signal transduction of hormones like insulin, FSH, and TSH [5].

In addition to MYO, other substances have demonstrated an ability to decrease insulin resistance and ameliorate metabolic dysfunction and inflammation. The positive effect of α-lipoic acid (ALA) on insulin resistance is now well recognized; indeed, researchers have demonstrated that either alone or in combination with MYO, ALA exerts multiple effects, including anti-inflammatory, antioxidant, and insulin-sensitizing ones, especially in overweight/obese patients [6,7,8].

Another compound that is known to positively interfere with blood glucose levels and ameliorate the management of comorbidities related to diabetes and its complications is phlorizin (PZN), which belongs to a class of dihydrochalcones. First isolated in 1835 from the bark of apple trees [9], PZN has the unique property of blocking glucose reabsorption through specific inhibitors of the sodium/glucose cotransporters (SGLTs) in the intestine (SGLT1) and the kidney (SGLT2). This blockage induces glycosuria by inhibiting renal reabsorption of glucose, and can be used as an adjuvant treatment for T2DM [10].

Another ingredient with a known positive effect in the treatment of metabolic dysregulations is the edible medicinal mushroom *Coprinus comatus* (CC), belonging to the family of *Agaricaceae*. CC has been extensively studied due to its numerous well-known pharmacological qualities; these include anti-obesity and antidiabetic properties, antioxidant and anti-inflammatory activities, and a potent hepatoprotective effect [11].

The amino acid glycine is a precursor to proteins. It is particularly involved in branched-chain amino acid (BCAA) and aromatic amino acid metabolism, contributing to the scavenging of excess acyl groups. Its role in obesity and/or insulin resistance is well known; indeed, researchers long ago demonstrated that an excess of BCAA is related to increased insulin resistance in obese patients [12].

Zinc is an essential micronutrient in human physiology; however, it cannot be stored in significant amounts, so regular dietary intake is crucial in maintaining adequate concentrations. Acquired zinc deficiency usually presents later in life, alongside risk factors like malabsorption syndromes; however, the use of medications like diuretics and angiotensin-receptor blockers can also cause zinc deficiency in younger individuals. Supplementation with this metal leads to amelioration of the immune system and attenuation of oxidative stress and inflammation while at the same time helping to maintain healthy gut microbiota [13].

Vitamin B^6^ is known as a molecule of interest in metabolic dysfunctions due to its potent anti-inflammatory and antioxidant properties. Deficiency in this vitamin is well established as a contributor to inflammatory-related conditions such as T2DM, obesity, and cardiovascular diseases. It is also known that B^6^ supplementation can reverse these inflammatory effects [14].

Chromium is an essential trace mineral that is usually combined with picolinic acid to enhance absorption. Chromium picolinate (CrP) is present in numerous foodstuffs, including whole grains, fruits, meat, and vegetables. CrP improves insulin resistance, glycemic control, and body composition in patients with altered metabolic status. These effects have been demonstrated in a number of published studies, as well as in systematic reviews and meta-analyses [15].

On the basis of the above-mentioned considerations, the aim of the present study was to evaluate the efficacy and safety of a combination of natural compounds (myo-inositol, α-lipoic acid, phlorizin, *Coprinus comatus*, glycine, zinc, vitamin B^6^, and chromium picolinate) which were used as a dietary supplement to aid the re-establishment of metabolic homeostasis in a group of overweight patients with documented insulin resistance. The key hypothesis underlying the present investigation was that this supplementation, acting either on metabolic parameters and/or on inflammation and oxidative status, would improve insulin and glucose differentials, decrease HOMA-IR index values, and ameliorate lipid profiles.

## 2. Materials and Methods

### 2.1. Study Design and Participants

The present open-label, multicenter, observational study was carried out between November 2021 and July 2023, and involved outpatients enrolled at various Italian medical centers, located in Rome, Milan, Bari, and Naples. All these centers are specifically engaged in the treatment of overweight, obesity, metabolic dysfunctions, and cardiovascular diseases. Approval from the Internal Review Board was obtained, and the study was conducted in line with Good Clinical Practice guidelines and the Declaration of Helsinki. Accordingly, all patients provided written informed consent.

A total of 75 patients (50 females; 25 males), aged 18–65 years (mean 46.1 ± 13), with BMI values > 25 kg/m^2^ (mean at T0 28.23 ± 2.58), fasting glucose levels between 110 and 125 mg/dL, and documented insulin resistance (HOMA-IR index > 2.5 and altered OGTT for insulin) were enrolled. Exclusion criteria were as follows: altered thyroid function; T2DM; addiction to alcohol and/or drugs; pregnancy or breastfeeding; previous treatments with drugs or supplements that might alter or modify the mechanism of action and/or the levels of insulin; patients with dermatitis and atopic predisposition (with or without their specific treatment); and patients with known fertility problems.

All patients underwent a preliminary check at enrollment (T0). This is described in greater detail in the next paragraph. Participants were then asked to begin treatment with a nutraceutical preparation containing myo-inositol (600 mg), glycine (250 mg), *Coprinus comatus* (200 mg), α-lipoic acid (200 mg), phlorizin (25 mg), zinc (10 mg), vitamin B6 (3 mg), and chromium picolinate (120 mcg) (Chromitron, Piemme Pharmatech, Rome, Italy). Two tablets were taken per day for the entire 3-month study period. Because there was no “Control group” in the present study, to better understand the effects of the above-mentioned combination, patients were asked not to modify their lifestyle in terms of diet and physical activity; these, therefore, remained unchanged throughout the observation period. Because some information exists, though very weak and based on animal studies, which suggests a possible negative impact of high doses of CrP on Leydig cells, every male patient of this study was subjected to testosterone and androstenedione measurements, before and after the observation period.

### 2.2. Measurements

At T0, each patient underwent a physical evaluation for anthropometric measures (weight, height, BMI, abdominal circumference). Blood samples were also collected at this time to confirm inclusion criteria (insulin at 0’ and +120’, blood glucose levels at 0’ and +120’, HOMA-IR index) and determine levels of total cholesterol, LDL, HDL, and triglycerides. To minimize patient discomfort, OGTTs for insulin and glucose involved collections at 0’ and +120’ timepoints only. Our method of ascertaining insulin and glucose status was thus easy, replicable, and convenient.

At T3, the same measurements were taken as at T0, and all data collected were subjected to appropriate statistical evaluation. In addition, patients were asked to immediately report the appearance of side effects at any level of intensity (low, mild, or strong) throughout the entire period.

After blood samples were collected, they were immediately centrifuged and stored (−20°) until assays were performed by the same laboratory using automated devices. With regard to measurements of blood glucose levels, in order to minimize glycolysis, samples were collected in separate vials containing sodium fluoride.

### 2.3. Statistical Analysis

Data series were checked for completeness; the database was then locked and validated before formal analysis was carried out. Continuous series were summarized in the form of mean and standard deviation with 95% confidence intervals (95% CIs), while discrete and binary series were summarized in the form of raw frequency and percentage. The distribution of continuous series was checked for normality using the Shapiro–Wilk test and by means of histograms. Longitudinal comparison of normally distributed series was carried out using Student’s *t*-test for paired data; for non-normally distributed series, the rank-sum test was used, adopting Fisher’s exact p correction. A *p*-value threshold for significance was set at 0.05, as per convention. A post hoc power analysis was carried out which showed that the total sample of 74 patients had a statistical power of 95% (alpha 0.05, Cohen’s D 0.76) for detecting significance in insulin changes in the OGTT curve. All computations were carried out using STATA software (ver. 18, Stata Corp, College Station, TX, USA).

## 3. Results

**Figure 1:**
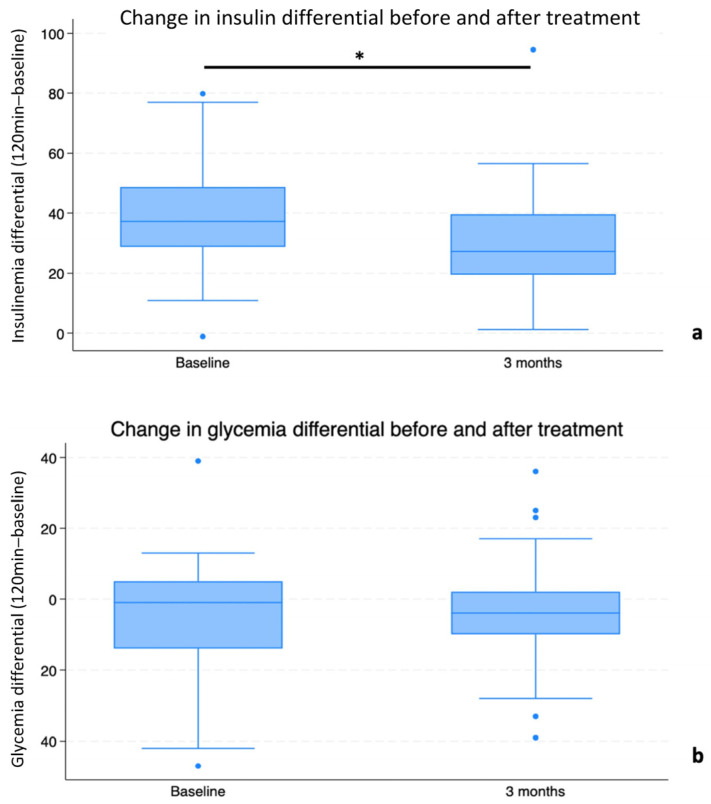
Differentials (deltas) between fasting blood insulin (**a**) and glucose (**b**), and their respective levels 120 min after 75 g oral glucose administration, at T0 and after a 3-month (T3) period of Chromitron administration. Data are presented in the form of mean ± SD for a group of 74 overweight patients with insulin resistance. * = *p* < 0.05.

Seventy-five patients entered the study; however, one male participant spontaneously decided to drop out immediately after enrollment. Therefore, a total of 74 patients (50 females, 24 males) entered the observation period. Differences were recorded in terms of amelioration of metabolic and anthropometric parameters. In particular, the results (expressed as mean ± SD and analyzed as described in the previous section) concerning the primary endpoints—first of all, the difference between basal insulin and blood levels after 120 min—revealed a significant decrease at the 3-month follow-up (∆ T0: 40.22 ± 1.96 μIU/mL vs. ∆ T3: 29.41 ± 1.84 μIU/mL; *p* < 0.00002). Concerning differences in glucose levels, while a similar reduction in the differential as that for insulin was documented at T3, this reduction did not reach statistical significance (∆ T0: 9.01 ± 1.84 mg/dL vs. ∆ T3: 6.46 ± 1.61 mg/dL; *p* < 0.544 = NS) (Figure 1a,b).

**Figure 2:**
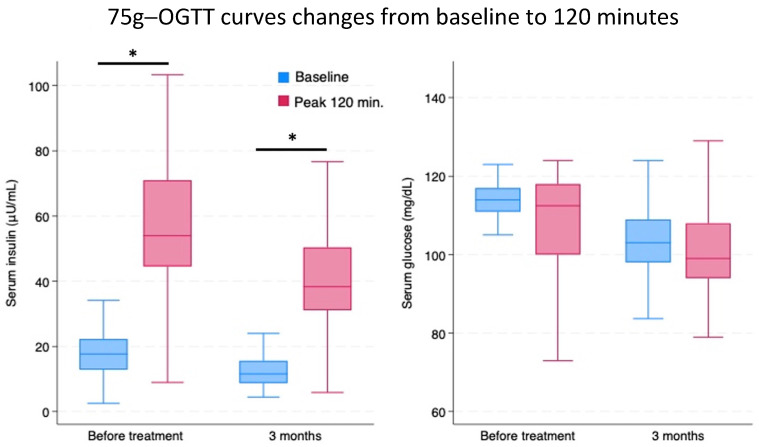
Insulin and glucose blood concentrations at baseline and at +120 min, after 75 g oral glucose administration, before and after a 3-month period of Chromitron administration (T3). Data are presented in the form of mean ± SD for a group of 74 overweight patients with insulin resistance. Blue columns represent baseline; pink columns represent +120 min. * = *p* < 0.05.

In addition to the deltas illustrated in Figure 1, to better characterize the variations in blood concentrations of insulin and glucose obtained from OGTTs at T0 and T3, Figure 2 and Table 1 present results in the form of raw data.

**Figure 3:**
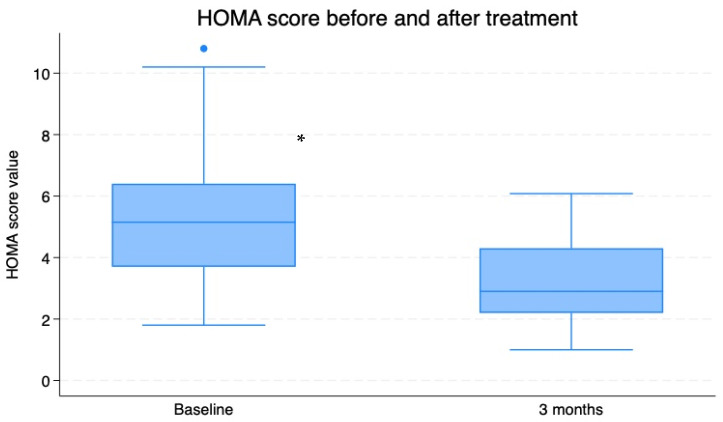
HOMA-IR index scores at T0 (baseline) and after a 3-month period of Chromitron administration (T3). Data are presented in the form of mean + SD for a group of 74 overweight patients with insulin resistance. * = *p* < 0.0001, baseline vs. T3.

As a consequence, in line with the amelioration of the metabolic parameters (insulin and glucose blood concentrations), the HOMA-IR levels significantly dropped as well (HOMA-IR at T0 = fasting glucose X fasting insulin/405: 5.31 ± 0.26 vs. T3: 3.19 ± 0.17; *p* < 0.0001; Figure 3).

**Figure 4:**
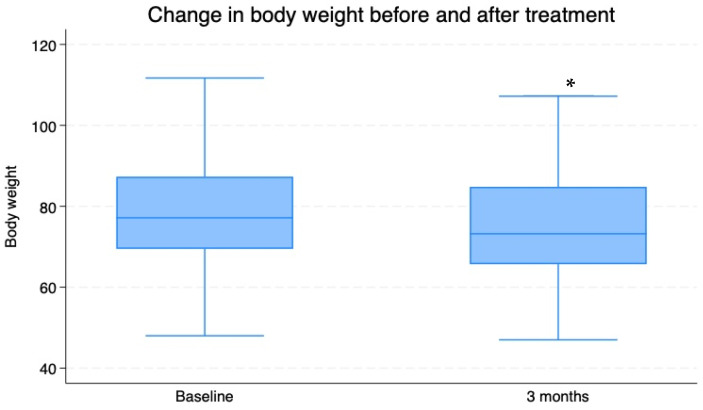
Mean ± SD values for body weight (kg) of 74 overweight patients with insulin resistance before (T0) and after a 3-month period of Chromitron administration (T3). * = *p* < 0.0001.

**Figure 5:**
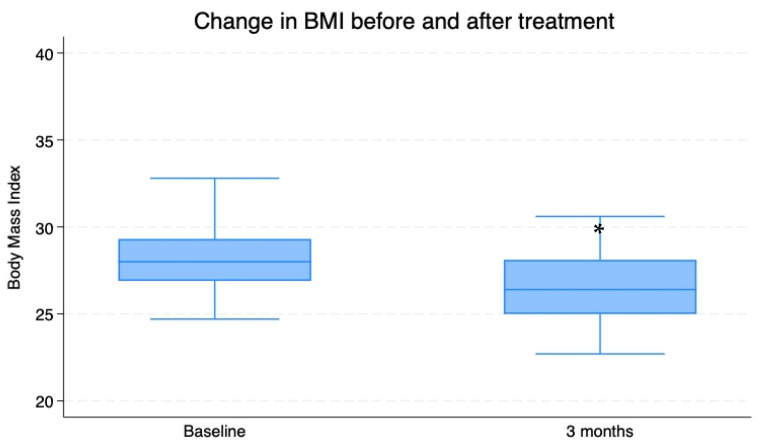
Mean ± SD values for BMI of 74 overweight patients with insulin resistance before (T0) and after a 3-month period of Chromitron administration (T3). * = *p* < 0.0001.

**Figure 6:**
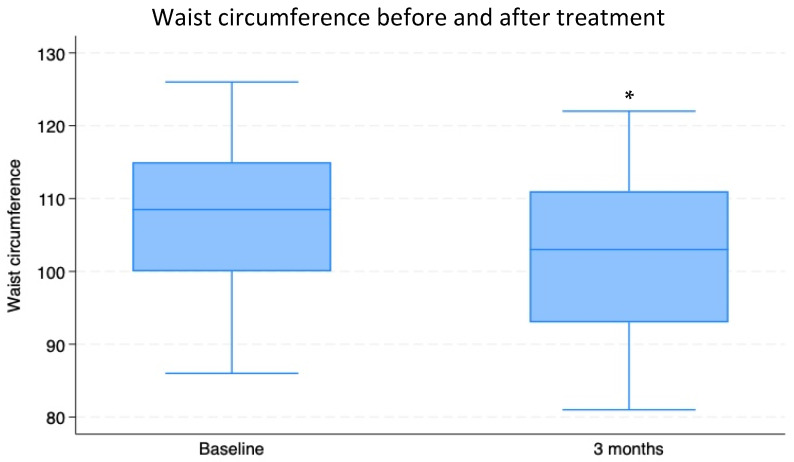
Abdominal circumferences (mean ± SD) of 74 overweight patients with insulin resistance before (T0) and after a 3-month period of Chromitron administration (T3). * = *p* < 0.0001.

During the observation period, a significant decrease in weight was also documented (weight at T0: 79.61 ± 0.44 kg vs. T3: 75.5 ± 1.46 kg; *p* < 0.009), which was associated with reductions in BMI (BMI at T0 = weight/height^2^: 28.23 ± 2.58 vs. T3: 26.7 ± 2.7) and abdominal circumference (AC at T0: 107.25 ± 1.18 cm vs. T3: 102.351 ± 1.26 cm; *p* < 0.002; Figure 4, Figure 5 and Figure 6).

**Figure 7:**
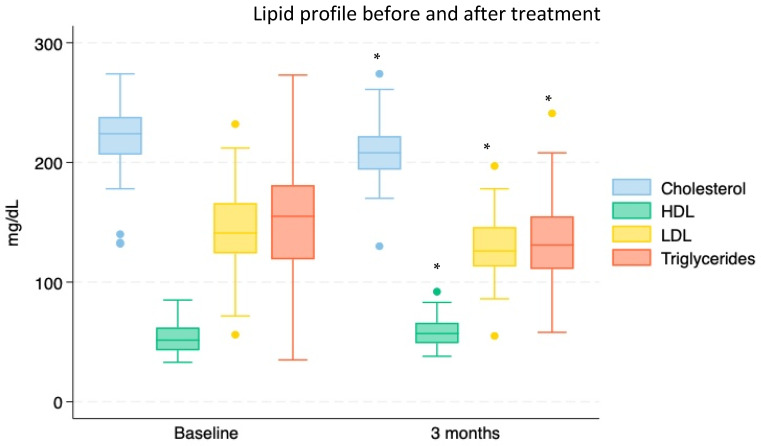
Blood levels of total cholesterol, HDL cholesterol, LDL cholesterol, and triglycerides at baseline (T0) and after a 3-month period of Chromitron administration (T3) in a group of 74 overweight and insulin-resistant patients. * = *p* < 0.03 and under.

Concerning secondary endpoints, our evaluations of cholesterol (total, LDL, and HDL) and triglycerides at T0 and after 3 months again revealed a significant amelioration of all the parameters that were considered (total cholesterol: 222.11 ± 3.39 mg/dL vs. 208.88 ± 2.85 mg/dL; *p* < 0.002—LDL cholesterol: 143.68 ± 3.84 mg/dL vs. 127.86 ± 3.14 mg/dL; *p* < 0.001—HDL cholesterol: 54.26 ± 1.52 mg/dL vs. 58.10 ± 1.40 mg/dL; *p* < 0.033—triglycerides: 152.26 ± 5.69 mg/dL vs. 134.35 ± 4.51 mg/dL; *p* < 0.008; Figure 7).

With regard to the safety of the combination, none of the 74 enrolled patients decided to discontinue the study due to the side effects of the product. A very low percentage of participants (0.4%) described the tablet as “difficult to swallow” due to its size. A similar proportion of subjects (0.5%) reported the presence of temporary gut discomfort, which did not require any intervention and disappeared in a few days.

Among male participants, testosterone and androstenedione assays before and after the observation period did not show any significant differences, thus indicating the safety of the CrP preparation used in the present study.

## 4. Discussion

The aim of the present observational study was to assess whether a combination of compounds, each known to positively interfere with metabolic alterations commonly present in overweight patients, could be efficaciously and safely used to ameliorate metabolic status in a group of overweight individuals. To this end, and as described with greater clarity in the “Results” section above, a number of overweight patients with confirmed insulin resistance were given a specific nutraceutical combination consisting of myo-inositol, α-lipoic Acid, phlorizin, *Coprinus comatus,* glycine, zinc, vitamin B^6^, and chromium picolinate, which is commonly available to patients (Chromitron, Piemme Pharma Tech, Italy). It is well known that these constituent substances each have their own specific effects on metabolic disturbances in insulin and glycemic control and/or in the area of inflammation and oxidative stress. Indeed, the specific roles played by each component are supported by a considerable number of scientific publications [1,3,10,16,17,18,19]. In particular, excessive weight induces either insulin resistance as a defense mechanism or a chronic inflammatory condition that self-maintains metabolic alterations. In the inositol family, myo-inositol (MYO) is converted to D-chiro-inositol (DCI) through the insulin-dependent activity of the enzyme epimerase [16]. In physiological conditions, epimerase converts MYO to DCI, according to the needs of the organism, in a tissue-specific ratio that is now well known. Specifically, MYO promotes glucose uptake, while DCI promotes glycogen storage [20]. Contrarily, if an altered metabolic condition is present, the MYO-to-DCI ratio is altered, creating a vicious circle that results in insulin resistance being maintained [21]. Data published very recently indicate that individuals with obesity-dependent gut microbiota are able to accelerate myo-inositol degradation, resulting in lower levels of MYO and an altered MYO/DCI ratio [22]. Reduction in weight resulting from the addition of MYO during interventions is therefore considered a milestone in the quest to reduce insulin resistance and chronic inflammation [23]. In another recent study, it was found that MYO improved hormonal and metabolic profiles in a group of PCOS patients [24]. In addition, when compared with metformin, MYO has been shown to exhibit a similar level of efficacy in the regulation of insulin resistance but with a better safety profile [17].

Another component present in the combination, and connected to inositols as far as metabolic function is concerned, was ALA. ALA is able to stimulate Phosphatidylinositol 3-Kinase (PI3K) activity and insulin receptor substrate phosphorylation in adipocytes. This involves the activation of additional intracellular mediators and the activation of GLUT4 translocation. For this reason, ALA can be considered an insulin mimetic agent [25].

Moreover, ALA is able to induce weight loss in rodents by reducing food intake and increasing energy expenditure [18]. With regard to its anti-inflammatory and antioxidant properties, human data demonstrate that ALA is able to increase the level of antioxidants and to reduce the level of peroxidation products, therefore reducing the deterioration of the oxidative system, thus decreasing the risk of obesity and cardiac dysfunction induced by high-fat diets [26].

As reported in the Introduction section, phlorizin, a plant compound with important pharmacological activities, exhibits a potent ability to block SGLT enzymes, thereby increasing glycosuria and reducing blood glucose levels. In addition, recent studies have demonstrated its significant antioxidant capacity and cyto-protective effects. These activities are exerted through the regulation of antioxidant enzyme activity, the inhibition of oxidative stress-related signaling pathways, and other mechanisms [27].

Among the compounds that were combined in the product used in the present study, *Coprinus comatus* seems to be the one with the widest range of positive effects on different organs. CC has been shown to prevent adipocyte differentiation through inhibition of PPARγ, the main regulator of adipocyte gene expression and differentiation. As a consequence, when given to obese rats, CC significantly reduced levels of fat mass, body weight, triglycerides, and total cholesterol concentrations, and increased levels of HDL cholesterol [28]. In addition to its anti-obesity effects, many studies have confirmed the antidiabetic activity of CC. In diabetic mice, this was found to be exerted through a reduction in hyperglycemia, the inhibition of gluconeogenesis, increases in glycogen and insulin, and the regeneration of injured β-cells, with decreased levels of glycosylated hemoglobin (HbA1C) also being reported [29,30]. In alloxan-induced diabetic rats, Comatin (a compound isolated from CC) was found to exert a more pronounced hypoglycemic effect than metformin, reducing levels of fasted blood glucose, postprandial blood glucose, and triglycerides [31,32]. Finally, recent studies have also suggested a relationship between CC and gut microbiota in which the hypoglycemic mechanism of CC is involved, at least in part [33].

The antioxidant and anti-inflammatory activities of CC have been extensively studied in animals. It has been demonstrated that polysaccharides from CC affect hepatic and mitochondrial antioxidant enzymes such as glutathione peroxidase (GSH-Px), superoxidedismutase (SOD), and catalase (CAT). As a consequence, treatment with polysaccharides from CC was found to increase the activity of hepatic GSH-Px by about 166.78%, SOD by about 83.72%, and CAT by about 63.12%. CC was also found to increase the activities of the mitochondrial enzymes GSH-Px, SOD, and CAT by about 92.00%, 67.03%, and 51.61%, respectively [34]. CC was also found to directly increase the levels of glutathione (GSH), when administered as an aqueous suspension to rats [35]. Similarly, CC has been shown to reduce proinflammatory factors, with reductions in tumor necrosis factor α (TNF-α), interleukin 1 beta (IL-1β), vascular endothelial growth factor alpha, and interleukin 17 (IL-17) of 58%, 27%, 47%, and 89%, respectively, being reported. Polysaccharides from CC have also been shown to significantly attenuate levels of interleukin 6 (IL-6), inducible nitric oxide synthase (iNOS), and cyclooxygenase 2 (COX-2), indicating that CC reduces inflammatory response caused by alcohol [34].

Regarding the potent hepato-protective effect of CC, reports have indicated that its polysaccharides are biologically active and, thus, may induce liver recovery after damage caused by alcohol consumption. In one animal study, it was shown that treatment with CC polysaccharide extract at a dose of 50 mg/kg b.w. could repair liver damage caused by alcohol [36], this being achieved through an increase in gut microbiota diversity and reductions in insulin resistance, hepatic inflammation, and oxidative stress [37]. Finally, because skin reactions in subjects with dermatitis and atopic predisposition were rarely reported during long-term CC assumption in some studies [11], patients with the same skin problems were excluded from the present study.

Vitamin B^6^ has been shown to downregulate genes associated with inflammatory and defense responses, such as cytokines, chemokines, and other inflammatory-related proteins. Supplementation with vitamin B^6^ was shown to suppress TNF-α and IL-6 levels in patients with rheumatoid arthritis by the authors of [19]. In addition, vitamin B^6^ supplementation has been shown to ameliorate some metabolic parameters in PCOS patients [38]. Moreover, when added to metformin as an adjuvant treatment, the bioactive derivative of vitamin B6, pyridoxal-5-phosphate, was found to improve blood glucose levels in patients with T2DM by the authors of [39].

Finally, it seems that the role played by chromium in metabolic dysfunction was of adjunctive benefit in the combination used in the present study. In one recent study of 122 subjects who continued with their normal physical and dietary habits, a statistically significant improvement in body composition was found after 90 days of supplementation with 400 mcg/day of chromium from CrP. CrP supplementation led not only to weight loss but also to favorable changes in body composition, as the weight lost was 98% fat mass and only 2% lean mass [40,41]. Therefore, because CrP appears to have such a “lean mass sparing effect”, it would seem advisable to include CrP as part of any reduced-calorie (diet) plan as a means of retaining metabolically active lean mass. Weight loss without CrP may be at the cost of lean mass; thus, this adjunctive strategy appears worthy of further research and applied practice [41]. In addition, a more recent review and meta-analysis showed that CrP is able to exert a direct antioxidant effect, increasing the total antioxidant capacity of the organism while indirectly stimulating glutathione production to a significant degree [42].

The amount of each component used in the combination was chosen according to previous studies reported in the literature [8,19,26,27,29,41,42]. In addition, because a control group was not used in our study, to confirm that any eventual variation in the considered parameters was due to the nutraceuticals used, patients were asked to not modify their own lifestyle during the observation period of 3 months.

As reported in the “Results” section, the 3 months of treatment induced a significant amelioration of insulin resistance, body weight, BMI, HOMA-IR, abdominal circumference, and blood glucose levels, though the latter did not reach statistical significance in our study, probably due to the number of patients. In addition, with regard to lipid profiles, we found that levels of total cholesterol, LDL cholesterol, and triglycerides significantly decreased after 3 months of treatment, while levels of HDL cholesterol increased, therefore indicating a decrease in cardiovascular risk. Moreover, side effects were minimal, both in terms of their incidence among patients and also in their degree of intensity, so none of the patients abandoned the study because of discomfort due to the nutraceutical product.

It is, therefore, possible to state that the active supplementation was able to ameliorate several parameters related to metabolic alterations and cardiovascular risk without appreciable side effects under the conditions of our study. Although the potential of these findings is exciting, a note of caution is due here due to certain limitations in our study. First, because this was an observational study and a control group was not present, it was impossible to ascertain whether patients changed their lifestyle during the observation period of 3 months, even though we told them not to do so. Therefore, the variable “unknown lifestyle changes” may have played a role in our results. In addition, even though, from a statistical point of view, the sample size was appropriate to evaluate the results, a group of 74 patients is not a significant sample size. Studies involving larger numbers of individuals are, therefore, required to confirm and expand upon our results. Moreover, even these results do not answer the question of whether the substances that were part of the combination acted synergistically, thus amplifying the total effect. Compared with their use alone, it can nonetheless be speculated that such a potent combination of substances, each of them acting on the same target, but from different facets, could exert a more potent effect when used together. Obviously, there is a need for more sophisticated and larger studies to unequivocally answer this question.

## 5. Conclusions

Despite these limitations, the results of this study indicate that a combination containing myo-inositol, α-lipoic acid, phlorizin, *Coprinus comatus,* glycine, zinc, vitamin B^6^, and chromium picolinate is an effective and safe tool that may be beneficial to patients who report an increase in weight and are affected by metabolic disturbances and/or increased cardiovascular risk associated with insulin resistance, pre-diabetes, high cholesterol, or excessive triglycerides. In addition, the use of this nutraceutical product could be of additional help when used as part of a multi-faceted and tailored protocol devoted to long-term weight control.

## Figures and Tables

**Table 1:** Summary data of insulin and glucose blood concentrations during OGTT test (mean ± SD).

Time	Insulin (μIU/mL)		Glucose (mg/dL)	
	Baseline	120’	Baseline	120’
T0	18.1 ± 7.1	58.2 ± 20.1	112.5 ± 8.4	107.6 ± 14.7
T3	12.4 ± 4.9	41.5 ± 17	103.4 ± 8.6	100.8 ± 11.4

## Data Availability

The datasets presented in this article are not readily available because the data are part of an ongoing study. Requests should be directed to M.N.
